# P257: Survey of the prevalence of healthcare associated infection at the Sahloul-Sousse teaching hospital-2010

**DOI:** 10.1186/2047-2994-2-S1-P257

**Published:** 2013-06-20

**Authors:** D Chebil, S Khefacha Aissa, H Said Latiri, M Ben Rejeb, N Jaidane, M Miladi, L Dhidah

**Affiliations:** 1 Microbiology Laboratory, Department of Hygiene and Service reanimation, Hospital of Sahloul, Sousse, Tunisia

## Introduction

The hospital hygiene service of the Sahloul Sousse Teaching Hospital (Tunisia) set up a surveillance system, based on regular prevalence surveys.

## Objectives

To monitor the prevalence of healthcare associated infections (HAI) in all services.

## Methods

The survey was carried in April 2010 to assess the prevalence of HAI and identify any risk factors. The data was collected on a given day for each hospital service and all services were monitored within the same week.

## Results

The survey concerned 352 patients with an average age of 48 ±24 years. 7.7% of patients had an infection, the prevalence of HAI being 38.5%. The intensive care services had the highest HAI rate followed by the surgical services and medical services. 42% of patients had a predisposition to infection. The most common extrinsic risk factors significantly related to HAI were parenteral nutrition, central venous catheters and urinary catheters. Pulmonary infections were the most common, followed by urinary tract infections, bacteremia and surgical site infections. The microorganisms most frequently implicated in the HAIs were Gram-negative bacteria (85%, n=21) including Enterobacter cloacae, Acinetobacter baumanii, Klebsiella pneumonia, Escherichia Coli and Pseudomonas aeruginosa.

## Conclusion

Monitoring HAI in this hospital helped to determine the priorities for corrective and preventive actions according to the problems identified.

## Competing interests

None declared

